# Exosomes: Emerging Therapy Delivery Tools and Biomarkers for Kidney Diseases

**DOI:** 10.1155/2021/7844455

**Published:** 2021-08-21

**Authors:** Can Jin, Peipei Wu, Linli Li, Wenrong Xu, Hui Qian

**Affiliations:** ^1^Key Laboratory of Laboratory Medicine of Jiangsu Province, School of Medicine, Jiangsu University, 301 Xuefu Road, Zhenjiang, Jiangsu 212013, China; ^2^Zhenjiang Key Laboratory of High Technology Research on Exosomes Foundation and Transformation Application, Jiangsu University, China

## Abstract

Exosomes are nanometer-sized small EVs coated with bilayer structure, which are released by prokaryotic and eukaryotic cells. Exosomes are rich in a variety of biologically active substances, such as proteins, nucleotides, and lipids. Exosomes are widely present in various body fluids and cell culture supernatants, and it mediates the physiological and pathological processes of the body through the shuttle of these active ingredients to target cells. In recent years, studies have shown that exosomes from a variety of cell sources can play a beneficial role in acute and chronic kidney disease. In particular, exosomes derived from mesenchymal stem cells have significant curative effects on the prevention and treatment of kidney disease in preclinical trials. Besides, some encapsulated substances are demonstrated to exert beneficial effects on various diseases, so they have attracted much attention. In addition, exosomes have extensive sources, stable biological activity, and good biocompatibility and are easy to store and transport; these advantages endow exosomes with superior diagnostic value. With the rapid development of liquid biopsy technology related to exosomes, the application of exosomes in the rapid diagnosis of kidney disease has become more prominent. In this review, the latest development of exosomes, including the biosynthesis process, the isolation and identification methods of exosomes are systematically summarized. The utilization of exosomes in diagnosis and their positive effects in the repair of kidney dysfunction are discussed, along with the specific mechanisms. This review is expected to be helpful for relevant studies and to provide insight into future applications in clinical practice.

## 1. Introduction

There are many types of kidney diseases. According to etiological factor, kidney diseases can be divided into primary, secondary, and congenital diseases. Acute kidney injury (AKI) produces various clinical manifestations accompanied with abrupt renal dysfunction. Nearly 20% of those hospitalized with AKI show increased resource utilization and poor outcomes. This prognosis correlates with many risk factors, including sepsis, trauma, diabetes mellitus, and older age [1, 2]. There are multiple factors that account for the mechanisms of AKI, including imbalanced inflammation, abnormal hemodynamics, and excessive production of reactive oxygen species (ROS) [3–5]. AKI and chronic kidney disease (CKD) are interconnected. Sustained pathological changes in AKI contribute to the development of CKD, which breaks the balance of the microenvironment among peritubular capillary, tubular cells, and interstitial cells. One of the most obvious features in CKD is the deposition of extracellular matrix and the formation of fibrosis [6]. Despite the existing diagnostic criteria (e.g., creatinine levels and urine output) and availability of treatments (e.g., dialysis, conservative care, and kidney transplantation), kidney diseases continue to pose a significant threat to people's health. Hence, the explorations of more sensitive biomarkers and more effective therapies are still two challenges to diagnose and treat kidney diseases.

Extracellular vesicles (EVs) generally fall into three categories: exosomes, microvesicles (MVs), and apoptotic bodies, which differ in size, origin, density, releasing mechanisms, and so on. MVs (100–1000 nm) are formed through the direct shedding of plasma membrane. Apoptotic bodies (100–5000 nm) are caused by cell apoptosis. Exosomes are formed through endosome systems and are nanoscale vesicles with diameters of 50–150 nm [7, 8]. It was analyzed that the protein patterns and lipidomes were differently enriched in exosomes and MVs even though they are released from the same cell source [9]. In 1983, exosomes were first identified in supernatants of sheep reticulocytes [10]. As research progressed, it was demonstrated that exosomes are secreted by the majority of cells (e.g., mesenchymal stem cells, macrophages, and cancer cells) [11–13] and widely distributed in biological liquids (e.g., plasma, urine, bone marrow, and amniotic fluid) [14–16] which play a crucial part in signaling transduction and pathological development of diseases [17, 18]. Because exosomes are present in body fluids, a strategy has been developed to enrich them and increase the feasibility and sensitivity of diagnostics [19]. As advances in engineered exosomes continue, many experiments have been conducted to improve their effectiveness. Xu et al. generated overexpression of circAkap7 exosomes via transfected circAkap7 into adipose-derived stromal cells. Exosomal circAkap7 functioned as a sponge to absorb miR-155-5p, enhanced autophagy, diminished oxidative stress, and relieved cerebral ischemic injury [20]. Wu et al. constructed engineered exosomes from human bone mesenchymal stem cells (hBMSCs) called mag-BMSC-Exos. They found that miR-21-5p was expressed more highly in mag-BMSC-Exos and inhibited Sprouty 2 (SPRY2) proteins and stimulated proangiogenesis and proproliferation pathways [21].

Many studies have reported therapeutic roles of exosomes secreted by cultured cells. Thanks to their reflection of host cells and existence in body fluids, studies have demonstrated the potential molecules which are associated with the diagnosis and prognosis of kidney diseases. This review summarizes those studies and provides insight into the possible use of exosomes.

## 2. Isolation, Identification, and Function of Exosomes

**Figure 1:**
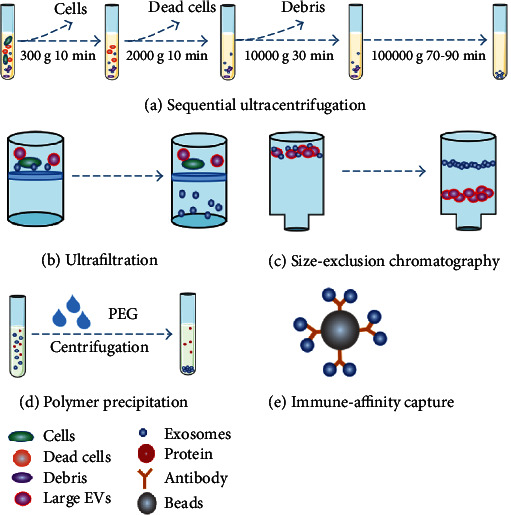
Five main traditional methods of exosome separation. Traditional methods are based on the physical, chemical, and biological properties of exosomes. Sequential ultracentrifugation is used to separate the exosomes, according to the different sedimentation coefficients of exosomes, cells, and debris. Ultrafiltration and size-exclusion chromatography depend on size: only exosomes can pass through a certain molecular-weight cut-off membrane and exhibit longer retention times in the stationary phase. When PEG is added, the surroundings are hydrophobic and promote deposition at the bottom. The beads are combined with specific antibodies to interact with the surface proteins of exosomes and can easily be sorted.

Exosomes are lipid-bilayer bioactive nanovesicles consisting of a multitude of proteins (e.g., heat-shock proteins, tetraspanins, and Alix), lipids (e.g., ceramide and cholesterol), and nucleic acids (e.g., DNA, mRNAs, and microRNAs). These bioactive molecules are partially involved in communication between cells and the modulation of recipient cells [22]. Various methods are traditionally used to isolate exosomes, such as sequential ultracentrifugation, ultrafiltration, size-exclusion chromatography, immune-affinity capture, and polymer precipitation (Figure 1). These methods depend on physical, chemical, and biological properties [23]. Ultracentrifugation is time-consuming, has high equipment requirement, and has no specificity. But it is suitable for large sample volumes. Comparatively, ultrafiltration is simple, fast, and low cost. However, exosomes will be trapped and clogged in the membrane and the isolated exosomes are still lack of specificity. Promisingly, sequential filtration is applied for large sample processing [24, 25]. Taking the advantages and disadvantages of each method into account, it is necessary to combine isolation techniques [24]. Because the extracted samples are not completely pure, and the biomarkers are not fully specific, Yang et al. proposed a mathematical formula to estimate the proportion of exosomes in a mixture [24]. Novel techniques have also been developed to overcome these drawbacks [23]. Lee et al. were the first to isolate exosomes with tangential flow filtration, which overcame the challenges of limited amounts of samples [26]. It is worth considering that the choice of methods differs depending on the sources of the sample. A previous study has found that differential ultracentrifugation was the most common method used for isolating exosomes from urine [27]. Sequential ultracentrifugation and polymer precipitation are often used for isolating serum exosomes. Nevertheless, sequential ultracentrifugation generates fewer particles, and polymer precipitation can cause clumping among the soluble proteins [28, 29]. Storage is another issue to consider. It is reported that exosomes degrade easily at 37°C. Therefore, they are suitable to be stored at -80°C while also need to be used as soon as possible [30].

Transmission electron microscopy (TEM), nanoparticle tracking analysis (NTA), flow cytometry (FCM), and Western blotting (WB) are common techniques for identifying concentration, size, and morphology [31]. Various methods have been suggested to characterize exosomes [32]. TEM assists in observing the shapes of exosomes visually and vividly. Their shapes are changeable depending on their surroundings, namely, whether they are cup-like or rounded [8, 33]. However, the instrument is expensive, the method is strict with the sample preparation, and the results are influenced by subjectivity [34]. NTA is used to measure the size distribution and concentration of exosomes [35]. Establishing the concentration makes it convenient to calculate the quantity of applied exosomes. Nevertheless, this technique is not sensitive, sorts out targeted exosomes with low efficiency, and lacks repeatability. FCM and WB are commonly used to identify molecular phenotyping [36, 37]. However, FCM outperforms in terms of its high throughput and accurate measurement of diameters and concentrations. To rigorously confirm the exosomes, different expressive abundances and functional markers should be selected, including CD9, CD63, CD81, Alix, calnexin, Grp94, and Tsg101 [36, 38]. In 2018, Théry et al. established guidelines for the isolation and characterization of EVs [32]. However, there remains a long way to go to standardize the quantity and quality of the extracted exosomes for experimental studies or clinical trials since inevitably obtaining non-EV composition.

Many studies have found that exosomes participate in cancer drug resistance [12], cutaneous wound healing [39], liver injury, and so on [40]. The cargos in EVs have been found to prevent cell death, modulate immune response, maintain vascular integrity, and promote cell activity [41]. Thus, attempts to recognize the underlying mechanisms of different EVs make it important for us to prevent this progression and seek means of addressing illness.

## 3. Diagnostic Roles of Circulating Exosomes in Kidney Diseases

Due to the insensitivity of traditional kidney injury biomarkers, advanced biomarkers are being sought and are rapidly emerging, such as kidney injury molecule-1, beta-2 microglobulin, cystatin C, and neutrophil gelatinase-associated lipocalin (NGAL). Nevertheless, researchers are still working to analyze an array of biomarkers to promote early diagnosis and treatment that may better represent relevant clinical manifestations and prognoses (Table 1) [42]. Blood and urine are two readily available specimens that can be obtained without invasive procedures or great pain to patients. miRNA is an important type of noncoding RNAs with about 22 nucleotides in length. It mainly exerted biological functions through posttranscriptional level. Differences in expression between healthy subjects and patients make it possible to be applied into diagnostic and theranostic markers [43]. Exosomes' structures protect their cargos (e.g., miRNA, circRNA, proteins, and lipids) from degrading and can represent the physiological or pathological states of their parental cells. With the development of methods to isolate exosomes from body fluid, promising potential to make use of bioactive molecules for prediction is being developed [14, 44].

### 3.1. Diagnostic Role of Urinary Exosomes

#### 3.1.1. Diagnosis of AKI

Because circulating exosomes rarely pass through the glomerular, the majority of urinal exosomes result from kidney, bladder, and prostate organs, which indicates their value for predicting certain diseases. Sonoda et al. established ischemia/reperfusion (I/R) models and conducted continuous observations on them for up to 2 weeks. It was demonstrated that miRNAs in exosomes could reflect the injury and fibrosis state, such as in the release of miR-9a, miR-16, miR-200a, and miR-141 [45]. Similarly, exosomal miR-30c-5p and miR-192-5p were confirmed to obviously increase both in animal models and in patients suffering from cardiovascular surgery [46]. It was also found that increased levels of organic anion transporter 5 (Oat5) were associated with aberrant renal function indexes in cisplatin-treated Wistar rats [67]. For sepsis-induced AKI, a specific transcriptional repressor for activating transcription factor 3 (ATF3) was increased but not detected in the non-AKI group [68]. Awdishu et al. conducted a survey among cirrhosis-induced AKI and showed that maltase glucoamylase increased significantly in urinary exosomes [69].

#### 3.1.2. Diagnosis of CKD

An overwhelming majority of scientists who have analyzed changeable microRNAs have used databases or profiling. Diabetic nephropathy is a serious complication caused by diabetes mellitus (DM). Using the results of bioinformatics analyses, Eissa et al. validated the high levels of miR-30a, miR-133b, and miR-342 in urine exosomes from type 2 diabetic nephropathy (T2DN) patients [50]. The use of these miRNAs was connected both with renal functions and with early diagnosis of albuminuria. Similar studies have shown that elevated miR-320c, which influences the TGF-*β*1 signaling pathway, could be used as a novel biomarker to distinguish microalbuminuria (MIC) from normoalbuminuria [48]. Accumulating studies also demonstrated different expressions for miR-192, miR-15b-5p, miR-let-7i-3p, miR-let-7c-5p, miR-24-3p, miR-27b-3p, and TGF-*β*1 in EVs [51, 53, 54]. Apart from this, multiple microRNAs have been explored as noninvasive predictors. miR-19b-3p, miR-15b, miR-34a, miR-150-5p, miR-362-3p, miR-636, and miR-877-3p increased in urinary exosomes from T2DN patients. This was speculated to be relevant to inflammation, proliferation, and apoptosis through regulating pathways [47, 49, 52]. After the administration of advanced glycation end-products (AGEs), TGF-*β*/Smad3 was activated in cultured podocytes and contributed to the release of the exosomal Elf3 protein, which was only detected in T2DN patients [70]. Additionally, increased levels of C-megalin were detected in urinary exosomes which also participated in regulating the quantity of exosomes [71]. Uromodulin mRNA in urinary EVs also represented the severity of the progression of renal disease in a sense, and Wilms' tumor 1 (WT1) mRNA reflected glomerular injury [72, 73]. Interestingly, in type 1 diabetic nephropathy (T1DN) rat models, miR-451-5p showed opposite trends in renal tissues and urine and was related to the protection against renal fibrosis [55]. Urinary exosomal miRNAs changed between normoalbuminuric and microalbuminuric diabetic patients and reflected the severity of pathological change. Those microalbuminuric patients exhibited increased amounts of miR-145 and miR-130a and decreased amounts of miR-155 and miR-424 [56]. Furthermore, the changeable levels of miR-21, miR-29c, miR-181a, miR-200b, and regucalcin protein were observed in CKD [74–78].

#### 3.1.3. Diagnosis in Other Kidney Diseases

Lupus nephritis (LN) is a complication that results from systemic lupus erythematosus and is a major risk factor for ESRD. Evidence has shown that glomerular miR-26a expression is higher in both mouse models and patients. However, exosomal miR-26a from urine displayed different consequences [57]. Tangtanatakul et al. later found that miR-let-7a showed obvious decreases during the active phase of LN, which could indicate disease progression [60]. It has been also reported that miR-21, miR-29c, and miR-150 were correlated with the degree of LN. Decreased levels of miR-29c and increased levels of miR-21 and miR-150 hastened the progression of fibrosis through the TGF-*β*/Smad3 pathway [58, 61]. Additionally, miR-146a was associated with the severity and activity of LN [59]. It should be noted that Garcia-Vives et al. surveyed the prognosis and clinical response of LN patients and found that responding patients secreted augmented levels of miR-135b-5p, miR-107, and miR-31-5p in their urinary exosomes. Those miRNAs inhibited hypoxia-inducible factor-1*α* (HIF-1*α*) in renal cells, which predicted better recovery and reduced inflammation [79].

Polycystic kidney disease (PKD) is a complex disease that is commonly found in the form of autosomal dominant PKD. Recent results have found apparent changes in the protein components in the exosomes. The activator of G protein signaling 3 (AGS3) was involved in the pathological progression of PKD. Enhanced levels of this protein were detected both in PCK rats and human samples [80]. Furthermore, studies have reported that exosomes overexpress envoplakin, villin 1, prominin 1, and the cellular repressor of E1A-stimulated genes 1 (CREG1), indicating aberrant morphology and proliferation in the cells [81, 82]. Polycystin-1 (PC-1), polycystin-2 (PC-2), transmembrane protein 2 (TMEM2), miR-30a-5p, and miR-194-5p are also relevant to diagnosis and monitoring [62, 83].

The incidence of renal cell carcinoma (RCC) is significantly higher in North America and in Western, Central, and Eastern Europe. RCC is usually accompanied by other diseases, such as hypertension, urinary stones, and diabetes. Prediction of this disease before the appearance of symptom phenotypes is extremely important [84]. Butz et al. showed increased levels of miR-150-5p and decreased levels of miR-126-3p in clear-cell RCC patients. A combination of miR-126-3p, miR-449a, and miR-34b-5p could improve diagnostic sensitivity [63]. Furthermore, abnormally elevated polymerase I and transcript release factor (PTRF) were found in patients' urinary exosomes, regulated by the EGFR/Akt pathway [85]. Higher levels of miR-204-5p were detected prior to progression for Xp11.2 translocation RCC [64].

Minimal change disease (MCD) is the most common disease to cause nephrotic syndrome and is characterized by podocytopathy. Compared with MCD, focal segmental glomerulosclerosis (FSGS) patients show poor prognosis which desires noninvasive biomarkers [86]. In a gesture toward conducting differential diagnosis between MCD and FSGS, researchers have used miR profiles that establish changes in miR-1225-5p, miR-1915, miR-663, miR-193a, and miR-155 [65, 87]. Among children exposed to idiopathic nephrotic syndrome (NS), miR-194-5p and miR-23b-3p have been found to be useful for monitoring urine protein levels and reflecting disease progression [66]. Bhayana et al. conducted a survey to predict radiation nephropathy and demonstrated increased levels of miR-1224 and miR-21 in leukemia patients pretreated with total-body irradiation [88].

### 3.2. Diagnostic Role of Plasmic Exosomes

Plasma is another source for liquid biopsies. Plasmic exosomes exist in the blood through cargo packing, trafficking, and secretion from host cells which could be used as biomarkers. Recently, Xie et al. made comparisons of bioinformatics results between plasma and plasmic exosomes and found incomplete consistency. Interestingly, it was speculated that plasmic exosomes could participate in the morbidity and progression of disease, as their cargos (e.g., miR-22-3p, miR-29b-3p, miR-30e-3p, miR-143-3p, and miR-770-5p) were related to significant pathways, such as extracellular matrix–receptor interaction and mucin type-O-glycan biosynthesis [89]. miR-146a is also implicated in monitoring kidney injury in animal models induced by cisplatin [90]. MCD has been observed to be associated with increased levels of miR-30, miR-34, and miR-342 in patients [65]. A survey was conducted of the enrollment of patients categorized by the severity of their disease. It was also shown that miR-21 could be used to distinguish interstitial fibrosis (IF) from tubular atrophy at higher grades [91]. In addition, Xiao et al. and Dias et al. reported different levels of miR-92a-1-5p, miR-301a-3p, miR-424-3p, miR-149-3p, and miR-1293 in RCC patients' plasmic exosomes relative to those of control groups [92, 93].

In summary, exosomal contents, such as proteins, miRNAs, and lipids in urine and plasma make it possible to predict the onset and progression of disease earlier and faster. However, some challenges hinder the translation into clinical practice. The methods to isolate exosomes need to be optimized and standardized to improve reproducibility. The sensitivity and specificity of encapsulated molecules need to be further analyzed and confirmed. Also, preanalytical factors should be taken into consideration and more patients need to be enrolled as well to increase the accuracy and reliability of the results. The use of exosomes in diagnosis is expected to have a bright future.

Exosomes can be obtained not only from body fluids but also from the supernatant of cultured cells, including MSCs. Our group previously demonstrated the significant roles of derived exosomes in alleviating inflammatory bowel disease [94], hepatic oxidant injury [95], and cutaneous injury [96]. The encapsulated substances are suggested to participate in maintaining regeneration and homeostasis. Notably, emerging reports have also found protective effects for derived exosomes in relieving kidney injury, which implies possible new treatment strategies.

## 4. Mesenchymal Stem Cell-Derived Exosomes in Kidney Injury Repair

MSCs have the unique capability for self-renewal and differentiation. MSCs can be obtained from several sources, including bone marrow [97], adipose tissue [98], amnion, chorion, and the umbilical cord [99]. Numerous trials have been conducted recently to investigate the validity and tolerance of MSC therapy [100–102]. Exosomes are paracrine forms of MSCs outperforming them in safety and immunity, and they consist of bioactive molecules [25]. The following sections focus on the progress of current research on the use of MSC-derived exosomes for kidney injury repair (Table 2).

### 4.1. Protective Roles on AKI

#### 4.1.1. I/R-Induced AKI

EVs isolated from the conditioned medium have been proven to be an important mediator of MSCs in renoprotection. Human Wharton's jelly mesenchymal stromal cell- (hWJMSC-) derived exosomes, which are abundant in miR-30, have been reported to alleviate mitochondrial fission by blocking the activation of dynamin-related protein 1 (DRP1), and miR-16 and miR-15 are speculated to target CX3CL1 to reduce inflammatory responses in their early stages [103, 104]. Further, exosomes from hucMSCs (hucMSC-Exos) are capable of effectively reinforcing the NRF2/ARE pathway, which result in the reduction of malondialdehyde (MDA) and 8-hydroxy-2′-deoxyguanosine (8-OHDG) [105]. Another group reported that hucMSC-Exos were able to enhance the ERK 1/2 pathway and promoted dedifferentiation by delivering hepatocyte growth factor (HGF) mRNA [106]. In addition, isolated microvesicles (MVs) that contain exosomes have been proven to depress the generation of ROS by regulating NADPH oxidase-2 (NOX2) proteins [123]. Notably, Zhao et al. reported the involvement of MSC-Exos in maintaining mitochondrial function. Both normal hucMSC-Exos and mouse BMMSC-Exos contained mitochondrial transcription factor A (TFAM) and mitochondrial DNA (mtDNA). These molecules were shuttled to tubular cells to alleviate mitochondrial dysfunction and reduce apoptosis and inflammation [124]. Recently, Cao et al. highlighted the important roles of miR-125b-5p enriched in exosomes. They found that the adhesive molecules on exosomes assisted in homing to injured areas and miR-125b-5p targeted p53 to inhibit apoptosis and promote tubular repair [111].

Furthermore, exosomes released from hBMSCs have also been reported to have beneficial effects. The miR-199a-3p they contain has been shown to target semaphoring 3A (Sema3A) and induce antiapoptosis [125]. Prestimulating bone marrow MSCs (BMMSCs) with melatonin has been found to prompt more efficient regeneration [126]. Mouse BMMSCs have been reported to transport miR-233, which directly suppresses NLR family-pyrin domain containing 3 from producing its protective effects [127]. Those exosomes have been shown to overexpress C-C motif chemokine receptor 2 (CCR2), which absorb circulating CCL2 and attenuate the extensive tissue inflammation induced by monocytes and macrophages [117].

It has also been shown that exosomes from human adipose-derived mesenchymal stem cells (hADMSCs) are enabled to alleviate acute injury or even chronic pathological changes by upregulating tubular Sox9, which is abolished when adding inhibitors [112]. In addition to this, ADMSCs and derived exosomes have been shown to alleviate injury *in vivo* better than other types [128].

Interestingly, Li et al. successfully isolated human urine-derived stem cells (hUSCs) from fresh human urine. They confirmed that hUSC-Exos migrated to and were incorporated into injured tubule cells instead of hUSCs. The highest-expression exosomal miR-146a-5p was screened and shown to target interleukin-1 receptor-associated kinase 1 (IRAK1) to decrease oxidative stress and reduce inflammation [114]. In another study, miR-216a-5p was found to target phosphatase and tensin homolog (PTEN) and had antiapoptotic effects [115]. Exosomes from human amnion epithelial cells (hAECs) were reported to protect against apoptosis and inflammation, which might be associated with the encapsulated proteins [129]. Several reports have also provided convincing evidence that progenitor cells in glomeruli, tubules, and renal arteries relieve kidney injury via paracrine action. MSCs in glomeruli (GI-MSCs) appear to reduce kidney damage more effectively through sorting miRNAs into EVs and influence cell communication and signal transduction [119]. In addition, EVs from human placenta-derived MSCs (hP-MSCs) have been demonstrated to transfer miR-200a-3p, which activates the Keap1-Nrf2 pathway and exhibits protection for mitochondrial functions. Cao et al. traced EVs with aggregation-induced emission luminogens. This material was further confirmed to provide higher resolution, a lower background, and better biocompatibility [116].

#### 4.1.2. Toxin-Induced AKI

Cisplatin is a kind of chemotherapeutic regimen that is used to treat cancer. Due to its detrimental effects on the kidney, scientists and clinicians have sought for possible methods to reverse those effects. Previous studies have demonstrated the considerable effects of exosomes in nephrotoxicity induced by cisplatin. In 2013, Zhou et al. found that hucMSCs were able to repair kidney damage through paracrine action by orderly regulating proliferation and apoptosis-related pathways [107]. Others have focused on the specific relationship between exosomes and autophagy. It has been clearly shown that hucMSC-Exos are capable of enhancing autophagy by depressing the mTOR signaling pathway, which is in negative parallel to the autophagy level [130]. 14-3-3*ζ* is the isoform of 14-3-3 proteins. Through liquid chromatography/mass spectrometry (LC/MS), it was analyzed that 14-3-3*ζ* was abundant in hucMSC-Exos [39]. Taking into account the transportation of 14-3-3*ζ* by exosomes, we also shed light on the possible mechanisms of intrinsic and delivered 14-3-3*ζ* through a combination of ATG16L, which resulted in the greater formation of autophagosome precursors, enhancing proliferated ability, resisting apoptosis, and favoring survival. Autophagy flux was blocked by the inhibitor 3-methyladenine (3-MA) [131, 132]. Intriguingly, Cao et al. concentrated on three-dimensional culture and demonstrated that three-dimensional-cultured exosomes from hucMSCs outperformed in terms of production, concentration, and efficiency [133]. It has also been shown that conditioned mediums from BMMSCs can repair disordered kidney structure, strengthen the kidney function, and diminish the inflammatory factors in gentamicin-induced AKI [134]. Insulin-like growth factor-1 receptor (IGF-1R) mRNA can be transferred from BMMSCs and facilitate epithelial cell proliferation, which is abolished by gene silencing [122, 135].

#### 4.1.3. Sepsis-Induced AKI

Sepsis is a complex disease that is characterized by aberrant responses to infection and that is accompanied by extremely serious complications, such as multiple organ dysfunction and septic shock. The kidney is particularly vulnerable to sepsis. To enhance the immunomodulatory ability of hucMSCs in sepsis, Song et al. prestimulated them with interleukin-1*β* (IL-1*β*). They found that IL-1*β*-pretreated MSCs continued to meet the basic definition of MSCs and mitigated the disorganization of kidney, lung, and liver with downregulated interleukin-6 (IL-6) and tumor necrosis factor-*α* (TNF-*α*). They also reported that more macrophages transformed to M2 phenotypes in lung and liver tissues subjected to pretreated MSCs. They thus found that exosomes derived from MSCs contained abundant miR-146a. When it was transferred to macrophages, it inhibited targeted proteins at the posttranscriptional level and fostered M2 polarization. Due to the lack of research on the kidney, the role of exosome miR-146a in renal injury needs further elucidation [136]. Our group recently showed that hucMSC-Exos affected the expression of miR-146b and targeted IRAK1 in tubular cells, which resulted in the inhibition of the nuclear translocation of NF-*κ*B and the production of inflammatory factors. Whether rare miR-146b can be detected in exosomes and which exosomal cargos affect miR-146b in kidneys deserve further exploration [108].

Because deacetylase sirtuin 1 (SIRT1) protein participates in orchestrating normal physiological processes in cells, it is clear that SIRT1 protein expression is upregulated in tissues associated with resistance to apoptosis and improves inflammatory responses under the treatment of ADMSC-exosomes [113]. It should be noted that some researchers have compared the efficiency of healthy and apoptotic ADMSC-derived exosomes in terms of circulating inflammatory levels, immune cells, and survival rate. Healthy ADMSC-derived exosomes outperformed others in their regulation of inflammatory reactions and oxidative stress [137]. Zhou et al. found that exosomes secreted by endothelial progenitor cells (EPCs) inhibited pathological changes in the kidney that were relevant to miR-126-5p and miR-126-3p [122].

### 4.2. Protective Roles on CKD

Fibrosis formation is an important pathological change in CKD. Unilateral ureteral obstruction (UUO) rat models could represent pathological changes in renal interstitium, so Liu et al. explored the protective molecular mechanisms of conditional medium (CM) in them. CM derived from hucMSCs has been reported to fight against oxidative stress, IF, and apoptosis [138]. Tubular epithelial cells undergoing epithelial-mesenchymal transition (EMT) are reported to participate in kidney fibrosis. During EMT, epithelial cells switch to mesenchymal phenotype change and secrete profibrotic factors and cytokines favoring the formation of fibrosis [139, 140]. Therefore, inhibition of inflammatory responses and EMT seemed to be favorable for slowing the development of fibrosis. It had received detailed demonstration that hucMSC-CM inhibited the TLR4/NF-*κ*B signaling pathway, reduced inflammatory cell infiltration, decreased *α*-smooth muscle actin (*α*-SMA), and upregulated E-cadherin in rats and in NRK-52E cells [109]. Their laboratory also showed that hucMSC-Exos could be internalized into HK-2 cells and could partly reverse the EMT phenomena induced by oxalate and calcium oxalate monohydrate [141]. Ji et al. highlighted the vital participation of Yes-associated protein (YAP) in the development of renal fibrosis. Promisingly, it was found that hucMSC-Exos delivered casein kinase 1*δ* (CK1*δ*) and *β*-transducin repeat-containing protein (*β*-TRCP) to degrade YAP protein expression, which apparently had antifibrotic impacts [110]. In addition, Chen et al. modified ADMSCs with glial cell line-derived neurotrophic factor (GDNF), which had the ability to augment the SIRT1/eNOS pathway and further promoted angiogenesis, as well as protecting against tubulointerstitial fibrosis [142]. miR-let-7c has been shown to target TGF-*β*1 and to alleviate renal fibrosis in adenine and streptozotocin- (STZ-) induced animal models [143]. Recently, that group found that transfected miR-let-7c was packed into human BMMSC-Exos and was transferred to impaired areas to fight irreversible fibrosis [144].

DM, a systemic metabolic disease, another common cause of CKD, is characterized by persistent hyperglycemia. Prolonged hyperglycemia leads to continuous changes in the vessels and glomerulus. Podocytes, an essential component of the glomerular filtration barrier, have been documented to undergo adverse transformation of structure and function. It has been elucidated that exosomal miR-486 is transported from ADMSCs to podocytes, inhibits the Smad1/mTOR signaling pathway, enhances autophagy flux, and has cytoprotective effects [118]. Similar results have been found for rat BMMSCs in type I DM induced by STZ [145]. In 2018, our group reported the underlying mechanisms by which hucMSC-Exos alleviated glycometabolic disorders. We demonstrated that hucMSC-Exos could promote the translocation of glucose transporters 4 (GLUT4) to enhance glucose uptake, activate the insulin/AKT pathway to increase insulin sensitivity, and change the quality and quantity of pancreatic *β*-cells [146]. Li et al. confirmed that exosomes inhibited the deposition of fibrosis-related proteins and myofibroblast transdifferentiation, as well as downregulating the proliferative abilities induced by the PI3K/AKT and MAPK signaling pathways [147]. In addition, BMMSC-CM has been reported to possess antiapoptotic, antifibrotic, and anti-inflammatory effects [148].

**Figure 2:**
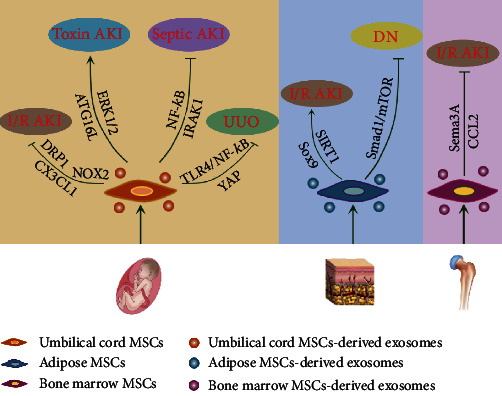
Mechanisms of MSC-exosomes in renal regeneration. MSCs derived from the umbilical cord, bone marrow, and adipose tissues play protective roles through antiapoptosis, anti-inflammation, antifibrosis, and antioxidation pathways.

Thus, a large amount of research has indicated that the exosomes of a range of stem cells protect against injury through antiapoptotic, anti-inflammatory, antioxidative, and antifibrotic pathways (Figure 2). The results of the studies reviewed also emphasize the roles of exosomal contents. However, some problems remain: it is not clear which pathway is the most significant, whether exosomes are harmful, and how to improve the effectiveness.

## 5. Other Cell-Derived Exosomes in Kidney Injury Repair

Hypoxia is a common cause of AKI. Under hypoxic circumstances, the exosomes secreted by tubular epithelial cells (TECs) are abundant in miR-20a-5p and are beneficial for the preservation of mitochondria and proliferation [149] (Table 2). In addition, exosomal ATF3 RNA protects against insult by downregulating monocyte chemotactic protein 1 (MCP1) and attenuating inflammatory responses [150]. When TECs are prestimulated with the appropriate hypoxia condition, more EVs are generated through the HIF-1*α*/Rab22 GTPase pathway, and they manifest more renoprotection [151]. In addition, renal proximal tubular cells (RPTCs) generate more protective exosomes during the early stages when they suffer from hypoxia [152]. These results demonstrate that adaptive injury to renal cells is advantageous to the kidney itself. Renal artery-derived vascular progenitor cells (RAPCs) in condition of oxidative stress are capable of promoting the migration of endothelial cells, which is related to increased Robo-1 and decreased miR-218 in exosomes [120]. Emerging evidence has shown the essential status that miR-486-5p has in endothelial colony-forming cell- (ECFC-) derived exosomes. Previously, it has been demonstrated that ECFC-exosomes protect against damage through their interaction between CXC chemokine receptor type 4 (CXCR4) and stromal cell-derived factor- (SDF-) 1*α* and thus transfer miR-486-5p [153]. Viñas et al. further illustrated the therapeutic mechanisms of miR-486-5p. ECFC-Exos contained obviously increased levels of miR-486-5p, which targeted PTEN and reinforced the Akt pathway to antagonize kidney injury [121]. It should be noted that exosomes from transfected macrophages also report apparent benefits during I/R injury [154, 155].

Recently, Grange et al. isolated EVs from urine characterized by aquaporin-1, aquaporin-2, and klotho, which demonstrated that the host was renal cells. The contents carried by EVs, such as miR-30, miR-151, and klotho, were shuttled to injured tissue, where they suppressed inflammation and promoted proliferation and recovery. However, those beneficial influences were reversed in klotho protein null mice, indicating the essential status of klotho protein [156]. Interestingly, Pan et al. later highlighted the importance of limb remote ischemic preconditioning in rescuing sepsis-induced AKI. They found a communication between skeletal muscle cells and tubule epithelial cells through circulation. They reported that myotube-derived and plasmic exosomes consisting of enhanced HIF-1*α*-dependent miR-21 depressed PDCD/NF-*κ*B, improved PTEN/Akt signaling pathways, and fought septic AKI [157].

These results indicate that cells interacted and contacted with each other through exosomes in the microenvironment. Exosomes from renal intrinsic cells or even distant cells have beneficial effects on promoting kidney regeneration, which sheds light on the functional mechanisms of other cells and provides another protective strategy.

## 6. Engineered Exosomes in Kidney Injury Repair

**Figure 3:**
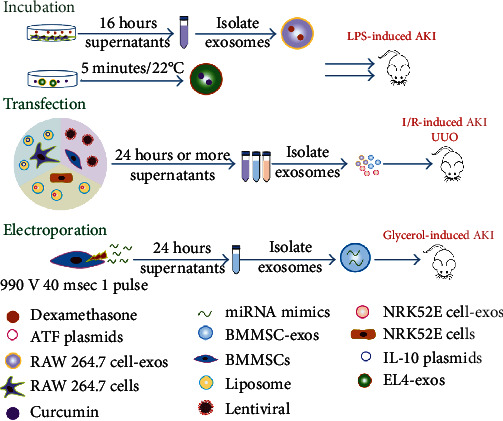
Methods for generating engineered exosomes in treating kidney injury. Incubation, electroporation, and transfection are three common methods of loading targeted molecules or substances into exosomes. These exosomes reveal benefits in anti-inflammatory, antiapoptotic, antioxidative, and antifibrotic aspects.

In spite of the essential roles that exosomes play in regeneration, their limited effectiveness still needs to be taken into consideration. Improving stability and targeting ability are two important issues for manufacturing engineered exosomes. Several studies have explored the impacts of engineered exosomes that are delivered through passive and active loading techniques. Passive loading includes incubation, and active loading includes electroporation, extrusion, sonication, and transfection [158]. Previous research demonstrated that corresponding ligand was expressed on the surface of exosomes through transfecting the host cells which improved targeting ability [30]. Notably, functional lipids (such as polyethylene glycol) were infused with exosomes and manifested better retention [159]. Besides, techniques such as bioconjugation, click chemistry, and hydrophobic insertion were also strategies to modify extracellular vesicles [158]. The evidence suggests a considerable status for kidney diseases (Figure 3).

Drug delivery is a promising strategy for treating AKI. BMMSCs incubated with melatonin have been reported to generate more protective exosomes during I/R-induced AKI [126]. In a study of septic shock, Sun et al. prepared curcumin-loaded exosomes by coincubating curcumin with EL-4-cell-derived exosomes. The mixture overcame the disadvantages of curcumin, namely, hydrophobicity, instability, and low bioavailability. They reported that the engineered exosomes exhibited obvious anti-inflammatory abilities and maintained curcumin bioactivity for a long period [160]. In another study, dexamethasone (DEX) and glucocorticoid receptor were packaged in the MVs of macrophages following incubation and extrusion. Integrin was found to be expressed on MVs and to be responsible for the target to kidneys. Compared to traditional glucocorticoid therapy, the engineered MVs had an edge in terms of sensitivity, effectiveness, and safety [161]. Furthermore, incubation has also been shown to change the bioactive substances in exosomes. Yoon et al. stimulated ADMSCs from healthy individuals using melatonin. They demonstrated that the stimulated exosomes inhibited senescence, preserved mitochondrial function, and enhanced proliferation of the ADMSCs from CKD patients. miR-4516 and cellular prion protein (PrP^C^) were found to be involved in the regeneration [162].

Transfection is also a strategy to edit the contents of the exosomes [150, 154]. Tang et al. obtained anti-inflammatory and engineered exosomes from M2 macrophages by transfecting IL-10 plasmids. They found that large amounts of IL-10 were loaded into exosomes, particularly with the administration of DEX. The exosomes maintained their bioactivity and promoted the targeting of interleukin, which inhibited mTOR signaling, induced M2 polarization, and promoted regeneration in an ischemic model [154]. In another study, miR-let-7c was transfected into hBMMSCs via a lentivirus. It was verified that exosomes were transferred into epithelial cells and downregulated fibrosis-related indexes such as TGF-*β*1, *α*-SMA, and collagen IV [144].

Tapparo et al. changed miRNA contents (miR-10a, miR-127, and miR-486) through electroporation and isolated corresponding exosomes. Notably, they found that the engineered exosomes were more effective at lower doses, and the overexpression of proregenerative miRNAs was not entirely beneficial [155].

Biochemical materials are also used to promote therapeutic efficacy. Intriguingly, Zhang et al. produced hydrogels containing RGD (Arg-Gly-Asp) peptide. RGD, integrin, EVs, and biotin were formed in an interacted network. Functionally, RGD increased the affinity to EVs, and hydrogels sustained the retention and stability. Mechanistically, EVs from hP-MSCs were found to contain high levels of miR-let-7a-5p, which targeted caspase 3 and RragD to reduce apoptosis and promote autophagy [163]. Similarly, the application of collagen matrix also showed beneficial effects on retention, and hP-MSCs-EVs reduced kidney injury through inhibiting endoplasmic reticulum stress [164].

Due to the low yield of exosomes or EVs, some researchers have concentrated on the function of nanovesicles (NVs). NVs are generated through the serial extrusion of hBMMSCs. Under the impacts of bacterial outer membrane vesicles, the administration of NVs has been reported to alleviate cytokine storm and increase levels of IL-10 in both conditioned media and serum [165].

Engineered exosomes indeed show supportive effects on kidney injury and even outperform other exosomes to some extent (Table 3). It is worth loading other protective molecules and combining them with other particles or biological materials. The research field of engineered exosomes is an emerging area between medicine and materials science and has promising prospects for application.

## 7. Conclusions and Prospects

In conclusion, due to the exploration of novel bioactive markers and the pursuit of noninvasive diagnosis, the cargos encapsulated in exosomes have garnered considerable research attention in their ability to promote early diagnosis and treatment and to relieve pain as soon as possible. Numerous studies have also confirmed that exosomes do indeed have regenerative effects. However, clinical treatment applications for exosomes in kidney diseases are still lacking.

It should be noted that exosomes also play an essential role in the pathological processes of kidney injury, shuttling from one cell to another. This phenomenon makes it possible and reasonable for us to prevent and treat severe injury by inhibiting the shuttling or downregulating adverse molecules [47, 166–173].

There have also been quite a few problems that hinder the development from laboratory research to clinical applications. Separation methods must be made efficient and rapid to meet the large number of patient specimens. The methods also need to be carefully selected. Different separation strategies best suited for different sampling resources [27]. In addition, more techniques are applied to enhance the specificity of exosomes. Purity is an important challenge that must be considered. So far, the coisolated contaminants inevitably exist in the exosomes. It is worth considering how the best method can be selected and accomplished with higher purity and lower contamination, such as with proteins and lipids. It is also suggested that purity can be measured through calculating the ratios, such as protein : particle ratio and protein : lipid ratio [32]. There is an urgent need to establish the standard of quantifying and evaluating exosomes, including the condition of host cells and potency tests. Notably, the purposes of basic and clinical research are different. Some non-EV components even show positive therapeutic effects [174].

In addition, the scale of production is not yet sufficient, which implies additional demands for improved cell culture protocols, novel isolation methods, and engineered exosomes [133, 144]. These are intended to increase the quantity of the product and improve its functional roles. Stimulation MSCs with bioglass ion products have been found to secrete more exosomes and to influence the contents. Increased levels of miR-1290 and decreased miR-342-5p have been shown to promote vascularization [175]. Multiple techniques have been used to modify exosomes in direct or indirect ways. Direct ways include loading molecules such as proteins, drugs, and mRNA into the isolated exosomes. Comparably, indirect ways are also used to transform cells and collect the corresponding exosomes afterwards [176]. In engineering, exosomes carry therapeutic substances without damaging the intrinsic properties [177]. Interestingly, Zhou et al. produced a dual-delivery biosystem in BMMSC-Exos through the electroporation of galectin-9 siRNA and vortex of oxaliplatin prodrug. It has been demonstrated that the modified exosomes show the ability to inhibit M2-like macrophage, activate CRT/HMGB1/ATP expression, and induce both innate and adaptive antitumor immune responses. This suggests a possible and promising strategy for treating melanoma. However, engineered exosomes remain at the exploratory stage, and it is not yet known which transformation is the most effective for certain models [178].

Furthermore, the biogenesis and ingredients of exosomes are not fully understood nor are their complex and long-term roles [179]. Exosomal biomarkers of diseases are still limited in the laboratory, and their reference range has not been established, which indicates that their standard of diagnosis is immature. The sensitivity and specificity of the indexes need to be more accurately detected and confirmed [93]. Through our persistent efforts, we hope to promote improvements of exosomes in tissue regeneration and to effectively alleviate clinical symptoms. Exosomes are expected to be a bright star in the future detection and treatment of diseases.

## Figures and Tables

**Table 1:** The diagnostic role of urine-derived exosomal miRNA in renal disease.

Kidney diseases	Changeable molecules	Mechanisms	Ref.
AKI	Upregulation: ↑ miR-200c, miR-9a, miR-141, miR-200a, miR-429	Target Zeb1/2 Regulate TGF-*β*-associated fibrosis	[45]
	miR-16, miR-24, miR-30c-5p, miR-192-5p	-	[45, 46]

T2DN	Upregulation: ↑ miR-19b-3p	Target SOCS-1 Regulate inflammation	[47]
	miR-320c	Target TSP-1 Regulate TGF-*β*-associated fibrosis	[48]
	miR-150-5p, miR-362-3p, miR-877-3p	Regulate p53, mTOR, AMPK pathways Involve in oxidative stress and fibrosis	[49]
	miR-133b, miR-30a, miR-342, miR-192, miR-15b, miR-34a, miR-636, miR-let-7c-5p	Regulate fibrosis	[50–53]
	Downregulation: ↓ miR-let-7i-3p, miR-24-3p, miR-27b-3p,	Involve in Wnt/*β*-catenin signaling	[54]

T1DN	Upregulation: ↑ miR-145, miR-30a Downregulation: ↓ miR-155, miR-424	-	[55, 56]

LN	Upregulation: ↑ miR-26a	Regulate podocyte differentiation and cytoskeletal integrity	[57]
	miR-21, miR-150	Target VEGFA and SP1 Regulate fibrosis	[58]
	miR-146a	Target IRAK1 and TRAF6 Regulate inflammation	[59]
	Downregulation: ↓ miR-29c, miR-let-7a	-	[60, 61]

PKD	Downregulation: ↓ miR-30a-5p, miR-30d-5p, miR-194-5p	-	[62]

RCC	Upregulation: ↑ miR-150-5p, miR-204-5p	-	[63, 64]
	Downregulation: ↓ miR-126-3p	-	[63]

FSGS	Upregulation: ↑ miR-155 Downregulation: ↓ miR-1915, miR-663	-	[65]

NS	Upregulation: ↑ miR-194-5p, miR-146b-5p, miR-378-3p, miR-23b-3p, miR-30a-5p	-	[66]

**Table 2:** The mechanisms of MSC and other cell-derived exosomes in attenuating kidney injury.

Sources of exosomes	Cargos	Species/models	Outcome	Ref.
hWJMSCs	miR-30	SD rats/left kidney ischemia for 45 mins	Inhibit DRP1 Antiapoptosis	[103]
	miR-15, miR-16	SD rats/left kidney ischemia for 60 mins	Inhibit CX3CL1 Anti-inflammation	[104]
	_	Rats/left kidney ischemia for 45 mins NRK-52E cells/hypoxia for 6 h	Activate Nrf2/ARE pathway Antioxidation	[105]

hucMSCs	HGF mRNA	SD rats/left kidney ischemia for 1 h	Activate ERK 1/2 pathway Antiapoptosis	[106]
	_	SD rats: 6 mg/kg cisplatin NRK-52E cells/5 *μ*M cisplatin for 6 h	Activate ERK 1/2 pathway Antiapoptosis	[107]
	_	C57BL/6 mice/CLP surgery	Inhibit NF-*κ*B pathway Anti-inflammation	[108]
	_	SD rats/unilateral ureteral obstruction NRK-52E cells/TGF-*β*1	Inhibit TLR4/NF-*κ*B pathway Anti-inflammation	[109]
	CK1*δ*, *β*-TRCP	SD rats/unilateral ureteral obstruction	Inhibit YAP activity Antifibrosis	[110]
	miR-125b-5p	C57BL/6 mice/bilateral kidneys ischemia for 30 mins HK-2 cells/hypoxia for 12 h	Inhibit p53 Antiapoptosis	[111]

hADMSCs	_	C57BL/6 mice/left kidney ischemia for 30 mins	Upregulate Sox9 Antifibrosis	[112]
	_	C57BL/6 mice/CLP surgery	Activate SIRT1 Anti-inflammation, Antiapoptosis	[113]

hUSCs	miR-146a-5p	SD rats/left kidney ischemia for 45 mins HK-2 cells/hypoxic medium 48 h	Downregulate IRAK1 Inhibit NF-*κ*B pathway Anti-inflammation	[114]
	miR-216a-5p	SD rats/left kidney ischemia for 45 mins HK-2 cells/hypoxic medium 1 h	Downregulate PTEN Antiapoptosis	[115]

hP-MSCs	miR-200a-3p	FVB mice/right kidney ischemia for 50 mins	Activated Keap1-Nrf2 pathway Antioxidation	[116]

Mouse BMMSCs	CCR2	BALB/c mice/left kidney ischemia for 1 h	Absorb CCL2 Anti-inflammation	[117]

Mouse ADMSCs	miR-486	C57BL/KsJ db/db mice	Inhibit Smad1/mTOR pathway Promote autophagy Antiapoptosis	[118]

GI-MSCs	miRNAs	SCID mice/left kidney ischemia for 35 mins	Promote proliferation	[119]

RAPCs	miR-218, Robo-1	C57BL/6 mice/bilateral kidney ischemia for 30 mins	Promote migration	[120]

ECFCs	miR-486-5p	FVB mice/bilateral kidney ischemia	Downregulate PTEN Activate Akt pathway	[121]

EPCs	miR-126-3p miR-126-5p	CD-1 outbred mice/CLP	Downregulate HMGB1 and VCAM1	[122]

**Table 3:** Engineered exosomes in kidney injury repair.

Sources of exosomes	Methods	Models	Outcome	Ref.
BMMSCs	Incubate with melatonin	I/R-induced AKI	Anti-inflammation, apoptosis, and oxidation Promote angiogenesis	[126]
	Transfect miR-let-7c lentiviral	UUO	Inhibit TGF-*β*1 Antifibrosis	[144]
	Electroporate miR-10a, miR-127, and miR-486	Glycerol-induced AKI	Promote regeneration	[155]

ADMSCs	Incubate with melatonin	Hind limb ischemia model with CKD	Antisenescence Preserve mitochondrial function Promote angiogenesis	[162]

hP-MSCs	Modify with RGD hydrogels	I/R-induced AKI	Antiapoptosis Promote autophagy	[163]
	Modify with collagen matrix	I/R-induced AKI	Inhibit endoplasmic reticulum stress Antiapoptosis and fibrosis Promote angiogenesis	[164]

EL-4 cells	Incubate with curcumin	LPS-induced AKI	Anti-inflammation	[160]

RAW 264.7 cells	Transfect IL-10 plasmid	I/R-induced AKI	Inhibit mTOR pathway Promote M2 macrophage polarization Anti-inflammation	[154]

RAW 264.7 cells	Incubate with dexamethasone	LPS-induced AKI Adriamycin-induced nephropathy	Anti-inflammation Antifibrosis Increase dexamethasone sensitivity	[161]

NRK-52E cells	Transfect ATF3 plasmid	I/R-induced AKI	Inhibit MCP-1 Anti-inflammation	[150]

## Abbreviations

AKI: Acute kidney injury
GFR: Glomerular filtration rate
KIM-1: Kidney injury marker-1
NGAL: Neutrophil gelatinase-associated lipocalin
ROS: Reactive oxygen species
CKD: Chronic kidney disease
ESRD: End-stage renal disease
EVs: Extracellular vesicles
hBMSCs: Human bone mesenchymal stem cells
SPRY2: Sprouty 2
MVs: Microvesicles
ILVs: Intraluminal vesicles
hucMSCs: Human umbilical cord mesenchymal stem cells
TEM: Transmission electron microscopy
NTA: Nanoparticle tracking analysis
FCM: Flow cytometry
WB: Western blotting
PEG: Hydrophobic polyethylene glycol
I/R: Ischemia/reperfusion
Oat5: Organic anion transporter 5
ATF3: Activating transcription factor 3
MGAM: Maltase glucoamylase
C-megalin: Full-length megalin
DM: Diabetes mellitus
T2DN: Type 2 diabetic nephropathy
MIC: Microalbuminuria
AGEs: Advanced glycation end-products
WT1: Wilms' tumor 1
T1DN: Type 1 diabetic nephropathy
LN: Lupus nephritis
HIF-1*α*: Hypoxia-inducible factor-1*α*
PKD: Polycystic kidney disease
AGS3: Activator of G protein signaling 3
CREG1: Cellular repressor of E1A-stimulated genes 1
PC-1: Polycystin-1
PC-2: Polycystin-2
TMEM2: Transmembrane protein 2
RCC: Renal cell carcinoma
PTRF: Transcript release factor
MCD: Minimal change disease
FSGS: Focal segmental glomerulosclerosis
NS: Nephrotic syndrome
IF: Interstitial fibrosis
Cd: Cadmium
MSCs: Mesenchymal stem cells
hWJMSCs: Human Wharton jelly mesenchymal stromal cells
DRP1: Dynamin-related protein 1
MDA: Malondialdehyde
8-OHDG: 8-Hydroxy-2′-deoxyguanosine
HGF: Hepatocyte growth factor
NOX2: NADPH oxidase-2 protein
TFAM: Mitochondrial transcription factor A
mtDNA: Mitochondrial DNA
Sema 3A: Semaphoring 3A
CCR2: C-C motif chemokine receptor 2
hADMSCs: Human adipose-derived mesenchymal stem cells
hUSCs: Human urine-derived stem cells
mucMSCs: Mouse umbilical cord mesenchymal stem cells
IRAK1: Interleukin-1 receptor-associated kinase 1
PTEN: Phosphatase and tensin homolog
hAECs: Human amnion epithelial cells
GI-MSCs: MSCs in glomeruli
hP-MSCs: Human placenta-derived MSCs
3-MA: 3-Methyladenine
IL-1*β*: Interleukin-1*β*
IL-6: Interleukin-6
TNF-*α*: Tumor necrosis factor-*α*
SIRT1: Sirtuin 1
EPCs: Endothelial progenitor cells
UUO: Unilateral ureteral obstruction
CM: Conditional medium
*α*-SMA: *α*-Smooth muscle actin
YAP: Yes-associated protein
CK1*δ*: Casein kinase 1*δ*
*β*-TRCP: *β*-Transducin repeat-containing protein
GDNF: Glial cell line-derived neurotrophic factor
STZ: Streptozotocin
GLUT4: Glucose transporters 4
TECs: Tubular epithelial cells
MCP1: Monocyte chemotactic protein 1
RPTCs: Renal proximal tubular cells
RAPCs: Renal artery-derived vascular progenitor cells
ECFC: Endothelial colony forming cell
CXCR4: CXC chemokine receptor type 4
CCL2: C-C motif ligand 2
SDF: Stromal cell-derived factor
DEX: Dexamethasone
mTOR: Mammalian target of rapamycin
NVs: Nanovesicles
NF-*κ*B: Nuclear factor-*κ*B
Shh: Sonic hedgehog
SOCS-1: Suppressors of cytokine signaling 1
TGF-*β*1: Transforming growth factor-*β*1
EMT: Epithelial-mesenchymal transition
PI3K: Phosphoinositide 3 kinase
Akt: Protein kinase B
Nrf2: Nuclear factor E2-related factor 2
ERK: Extracellular-signal-related kinase
CLP: Cecal ligation and puncture
TLR4: Toll-like receptor 4
PI3K: Phosphoinositide 3 kinase
AGEs: Advanced glycation end-products
PrP^C^: Cellular prion protein.

## References

[1] Levey A. S., James M. T. (2017). Acute kidney injury. *Annals of Internal Medicine*.

[2] Zeng X., McMahon G. M., Brunelli S. M., Bates D. W., Waikar S. S. (2014). Incidence, outcomes, and comparisons across definitions of AKI in hospitalized individuals. *Clinical Journal of the American Society of Nephrology*.

[3] Bellomo R., Kellum J. A., Ronco C. (2017). Acute kidney injury in sepsis. *Intensive Care Medicine*.

[4] Petejova N., Martinek A., Zadrazil J., Teplan V. (2019). Acute toxic kidney injury. *Renal Failure*.

[5] He L., Wei Q., Liu J. (2017). AKI on CKD: heightened injury, suppressed repair, and the underlying mechanisms. *Kidney International*.

[6] Tan R. J., Zhou D., Liu Y. (2016). Signaling crosstalk between tubular epithelial cells and interstitial fibroblasts after kidney injury. *Kidney diseases*.

[7] Mathieu M., Martin-Jaular L., Lavieu G., Théry C. (2019). Specificities of secretion and uptake of exosomes and other extracellular vesicles for cell-to-cell communication. *Nature Cell Biology*.

[8] Zhang Y., Liu Y., Liu H., Tang W. H. (2019). Exosomes: biogenesis, biologic function and clinical potential. *Cell & Bioscience*.

[9] Haraszti R. A., Didiot M. C., Sapp E. (2016). High-resolution proteomic and lipidomic analysis of exosomes and microvesicles from different cell sources. *Journal of Extracellular Vesicles*.

[10] Pan B. T., Johnstone R. M. (1983). Fate of the transferrin receptor during maturation of sheep reticulocytes in vitro: selective externalization of the receptor. *Cell*.

[11] Zhang B., Shen L., Shi H. (2016). Exosomes from human umbilical cord mesenchymal stem cells: identification, purification, and biological characteristics. *Stem Cells International*.

[12] Zheng P., Chen L., Yuan X. (2017). Exosomal transfer of tumor-associated macrophage-derived miR-21 confers cisplatin resistance in gastric cancer cells. *Journal of Experimental & Clinical Cancer Research*.

[13] Harada T., Yamamoto H., Kishida S. (2017). Wnt5b-associated exosomes promote cancer cell migration and proliferation. *Cancer Science*.

[14] Baranyai T., Herczeg K., Onódi Z. (2015). Isolation of exosomes from blood plasma: qualitative and quantitative comparison of ultracentrifugation and size exclusion chromatography methods. *PLoS One*.

[15] Street J. M., Koritzinsky E. H., Glispie D. M., Yuen P. S. T. (2017). Urine exosome isolation and characterization. *Methods in Molecular Biology*.

[16] Tracy S. A., Ahmed A., Tigges J. C. (2019). A comparison of clinically relevant sources of mesenchymal stem cell-derived exosomes: bone marrow and amniotic fluid. *Journal of Pediatric Surgery*.

[17] Xia W., Chen H., Chen D., Ye Y., Xie C., Hou M. (2020). PD-1 inhibitor inducing exosomal miR-34a-5p expression mediates the cross talk between cardiomyocyte and macrophage in immune checkpoint inhibitor-related cardiac dysfunction. *Journal for Immunotherapy of Cancer*.

[18] Ono K., Sogawa C., Kawai H. (2020). Triple knockdown of CDC37, HSP90-alpha and HSP90-beta diminishes extracellular vesicles-driven malignancy events and macrophage M2 polarization in oral cancer. *Journal of Extracellular Vesicles*.

[19] Wang Y., Li Q., Shi H. (2020). Microfluidic Raman biochip detection of exosomes: a promising tool for prostate cancer diagnosis. *Lab Chip*.

[20] Xu L., Ji H., Jiang Y. (2020). Exosomes derived from CircAkap7-modified adipose-derived mesenchymal stem cells protect against cerebral ischemic injury. *Frontiers in Cell and Development Biology*.

[21] Wu D., Kang L., Tian J. (2020). Exosomes derived from bone mesenchymal stem cells with the stimulation of Fe_3_O_4_ nanoparticles and static magnetic field enhance wound healing through upregulated miR-21-5p. *International Journal of Nanomedicine*.

[22] Rashed M. H., Bayraktar E., Helal G. K. (2017). Exosomes: from garbage bins to promising therapeutic targets. *International Journal of Molecular Sciences*.

[23] Gandham S., Su X., Wood J. (2020). Technologies and standardization in research on extracellular vesicles. *Trends in Biotechnology*.

[24] Yang D., Zhang W., Zhang H. (2020). Progress, opportunity, and perspective on exosome isolation - efforts for efficient exosome-based theranostics. *Theranostics*.

[25] Nikfarjam S., Rezaie J., Zolbanin N. M., Jafari R. (2020). Mesenchymal stem cell derived-exosomes: a modern approach in translational medicine. *Journal of Translational Medicine*.

[26] Lee J. H., Ha D. H., Go H. K. (2020). Reproducible large-scale isolation of exosomes from adipose tissue-derived mesenchymal stem/stromal cells and their application in acute kidney injury. *International Journal of Molecular Sciences*.

[27] EV-TRACK Consortium, van Deun J., Mestdagh P. (2017). EV-TRACK: transparent reporting and centralizing knowledge in extracellular vesicle research. *Nature Methods*.

[28] Coumans F. A. W., Brisson A. R., Buzas E. I. (2017). Methodological guidelines to study extracellular vesicles. *Circulation Research*.

[29] Jung H. H., Kim J. Y., Lim J. E., Im Y. H. (2020). Cytokine profiling in serum-derived exosomes isolated by different methods. *Scientific Reports*.

[30] Vakhshiteh F., Atyabi F., Ostad S. N. (2019). Mesenchymal stem cell exosomes: a two-edged sword in cancer therapy. *International Journal of Nanomedicine*.

[31] Gardiner C., Di Vizio D., Sahoo S. (2016). Techniques used for the isolation and characterization of extracellular vesicles: results of a worldwide survey. *Journal of Extracellular Vesicles*.

[32] Théry C., Witwer K. W., Aikawa E. (2018). Minimal information for studies of extracellular vesicles 2018 (MISEV2018): a position statement of the International Society for Extracellular Vesicles and update of the MISEV2014 guidelines. *Journal of Extracellular Vesicles*.

[33] Wu X., Showiheen S. A. A., Sun A. R. (2019). Exosomes extraction and identification. *Methods in Molecular Biology*.

[34] Cizmar P., Yuana Y. (2017). Detection and characterization of extracellular vesicles by transmission and cryo-transmission electron microscopy. *Methods in Molecular Biology*.

[35] Gurunathan S., Kang M. H., Jeyaraj M., Qasim M., Kim J. H. (2019). Review of the isolation, characterization, biological function, and multifarious therapeutic approaches of exosomes. *Cell*.

[36] Welsh J. A., van der Pol E., Bettin B. A. (2020). Towards defining reference materials for measuring extracellular vesicle refractive index, epitope abundance, size and concentration. *Journal of Extracellular Vesicles*.

[37] Lobb R. J., Becker M., Wen Wen S. (2015). Optimized exosome isolation protocol for cell culture supernatant and human plasma. *Journal of Extracellular Vesicles*.

[38] Théry C., Amigorena S., Raposo G., Clayton A. (2006). Isolation and characterization of exosomes from cell culture supernatants and biological fluids. *Current Protocols in Cell Biology*.

[39] Zhang B., Shi Y., Gong A. (2016). HucMSC exosome-delivered 14-3-3*ζ* orchestrates self-control of the Wnt response via modulation of YAP during cutaneous regeneration. *Stem Cells*.

[40] Jiang W., Tan Y., Cai M. (2018). Human Umbilical Cord MSC-Derived Exosomes Suppress the Development of CCl4-Induced Liver Injury through Antioxidant Effect. *Stem Cells International*.

[41] Roefs M. T., Sluijter J. P. G., Vader P. (2020). Extracellular vesicle-associated proteins in tissue repair. *Trends in Cell Biology*.

[42] Griffin B. R., Faubel S., Edelstein C. L. (2019). Biomarkers of drug-induced kidney toxicity. *Therapeutic Drug Monitoring*.

[43] Novák J., Macháčková T., Krejčí J., Bienertová-Vašků J., Slabý O. (2021). MicroRNAs as theranostic markers in cardiac allograft transplantation: from murine models to clinical practice. *Theranostics*.

[44] Svenningsen P., Sabaratnam R., Jensen B. L. (2020). Urinary extracellular vesicles: origin, role as intercellular messengers and biomarkers; efficient sorting and potential treatment options. *Acta Physiologica*.

[45] Sonoda H., Lee B. R., Park K. H. (2019). miRNA profiling of urinary exosomes to assess the progression of acute kidney injury. *Scientific Reports*.

[46] Zou Y. F., Wen D., Zhao Q. (2017). Urinary microRNA-30c-5p and microRNA-192-5p as potential biomarkers of ischemia-reperfusion-induced kidney injury. *Experimental Biology and Medicine*.

[47] Lv L. L., Feng Y., Wu M. (2020). Exosomal miRNA-19b-3p of tubular epithelial cells promotes M1 macrophage activation in kidney injury. *Cell Death and Differentiation*.

[48] Delić D., Eisele C., Schmid R. (2016). Urinary exosomal miRNA signature in type II diabetic nephropathy patients. *PLoS One*.

[49] Xie Y., Jia Y., Cuihua X., Hu F., Xue M., Xue Y. (2017). Urinary exosomal microRNA profiling in incipient type 2 diabetic kidney disease. *Journal Diabetes Research*.

[50] Eissa S., Matboli M., Bekhet M. M. (2016). Clinical verification of a novel urinary microRNA panal: 133b, -342 and -30 as biomarkers for diabetic nephropathy identified by bioinformatics analysis. *Biomedicine & Pharmacotherapy*.

[51] Jia Y., Guan M., Zheng Z. (2016). miRNAs in Urine Extracellular Vesicles as Predictors of Early-Stage Diabetic Nephropathy. *Diabetes Research*.

[52] Eissa S., Matboli M., Aboushahba R., Bekhet M. M., Soliman Y. (2016). Urinary exosomal microRNA panel unravels novel biomarkers for diagnosis of type 2 diabetic kidney disease. *Journal of Diabetes and its Complications*.

[53] Li W., Yang S., Qiao R., Zhang J. (2018). Potential value of urinary exosome-derived let-7c-5p in the diagnosis and progression of type II diabetic nephropathy. *Clinical Laboratory*.

[54] Prabu P., Rome S., Sathishkumar C. (2019). MicroRNAs from urinary extracellular vesicles are non-invasive early biomarkers of diabetic nephropathy in type 2 diabetes patients with the 'Asian Indian phenotype'. *Diabetes & Metabolism*.

[55] Mohan A., Singh R. S., Kumari M. (2016). Urinary exosomal microRNA-451-5p is a potential early biomarker of diabetic nephropathy in rats. *PLoS One*.

[56] Barutta F., Tricarico M., Corbelli A. (2013). Urinary exosomal microRNAs in incipient diabetic nephropathy. *PLoS One*.

[57] Ichii O., Otsuka-Kanazawa S., Horino T. (2014). Decreased miR-26a expression correlates with the progression of podocyte injury in autoimmune glomerulonephritis. *PLoS One*.

[58] Solé C., Moliné T., Vidal M., Ordi-Ros J., Cortés-Hernández J. (2019). An exosomal urinary miRNA signature for early diagnosis of renal fibrosis in lupus nephritis. *Cell*.

[59] Perez-Hernandez J., Martinez-Arroyo O., Ortega A. (2020). Urinary exosomal miR-146a as a marker of albuminuria, activity changes and disease flares in lupus nephritis. *Journal of Nephrology*.

[60] Tangtanatakul P., Klinchanhom S., Sodsai P. (2019). Down-regulation of let-7a and miR-21 in urine exosomes from lupus nephritis patients during disease flare. *Asian Pacific Journal of Allergy and Immunology*.

[61] Solé C., Cortés-Hernández J., Felip M. L., Vidal M., Ordi-Ros J. (2015). miR-29c in urinary exosomes as predictor of early renal fibrosis in lupus nephritis. *Nephrology Dialysis Transplantation*.

[62] Magayr T. A., Song X., Streets A. J. (2020). Global microRNA profiling in human urinary exosomes reveals novel disease biomarkers and cellular pathways for autosomal dominant polycystic kidney disease. *Kidney International*.

[63] Butz H., Nofech-Mozes R., Ding Q. (2016). Exosomal microRNAs are diagnostic biomarkers and can mediate cell-cell communication in renal cell carcinoma. *European Urology Focus*.

[64] Kurahashi R., Kadomatsu T., Baba M. (2019). MicroRNA-204-5p: a novel candidate urinary biomarker of Xp11.2 translocation renal cell carcinoma. *Cancer Science*.

[65] Ramezani A., Devaney J. M., Cohen S. (2015). Circulating and urinary microRNA profile in focal segmental glomerulosclerosis: a pilot study. *European Journal of Clinical Investigation*.

[66] Chen T., Wang C., Yu H. (2019). Increased urinary exosomal microRNAs in children with idiopathic nephrotic syndrome. *eBioMedicine*.

[67] Bulacio R. P., Anzai N., Ouchi M., Torres A. M. (2015). Organic anion transporter 5 (Oat5) urinary excretion is a specific biomarker of kidney injury: evaluation of urinary excretion of exosomal Oat5 after N-acetylcysteine prevention of cisplatin induced nephrotoxicity. *Chemical Research in Toxicology*.

[68] Panich T., Chancharoenthana W., Somparn P., Issara-Amphorn J., Hirankarn N., Leelahavanichkul A. (2017). Urinary exosomal activating transcriptional factor 3 as the early diagnostic biomarker for sepsis-induced acute kidney injury. *BMC Nephrology*.

[69] Awdishu L., Tsunoda S., Pearlman M. (2019). Identification of maltase glucoamylase as a biomarker of acute kidney injury in patients with cirrhosis. *Critical Care Research and Practice*.

[70] Sakurai A., Ono H., Ochi A. (2019). Involvement of Elf3 on Smad3 activation-dependent injuries in podocytes and excretion of urinary exosome in diabetic nephropathy. *PLoS One*.

[71] de S., Kuwahara S., Hosojima M. (2017). Exocytosis-mediated urinary full-length megalin excretion is linked with the pathogenesis of diabetic nephropathy. *Diabetes*.

[72] Yamamoto C. M., Murakami T., Oakes M. L. (2018). Uromodulin mRNA from urinary extracellular vesicles correlate to kidney function decline in type 2 diabetes mellitus. *American Journal of Nephrology*.

[73] Abe H., Sakurai A., Ono H. (2018). Urinary exosomal mRNA of WT1 as diagnostic and prognostic biomarker for diabetic nephropathy. *The Journal of Medical Investigation*.

[74] Lv C. Y., Ding W. J., Wang Y. L. (2018). A PEG-based method for the isolation of urinary exosomes and its application in renal fibrosis diagnostics using cargo miR-29c and miR-21 analysis. *International Urology and Nephrology*.

[75] Kumari M., Mohan A., Ecelbarger C. M., Gupta A., Prasad N., Tiwari S. (2020). miR-451 loaded exosomes are released by the renal cells in response to injury and associated with reduced kidney function in human. *Frontiers in Physiology*.

[76] Khurana R., Ranches G., Schafferer S. (2017). Identification of urinary exosomal noncoding RNAs as novel biomarkers in chronic kidney disease. *RNA*.

[77] Zubiri I., Posada-Ayala M., Benito-Martin A. (2015). Kidney tissue proteomics reveals regucalcin downregulation in response to diabetic nephropathy with reflection in urinary exosomes. *Translational Research*.

[78] Yu Y., Bai F., Qin N. (2018). Non-proximal renal tubule-derived urinary exosomal miR-200b as a biomarker of renal fibrosis. *Nephron*.

[79] Garcia-Vives E., Solé C., Moliné T. (2020). The urinary exosomal miRNA expression profile is predictive of clinical response in lupus nephritis. *International Journal of Molecular Sciences*.

[80] Keri K. C., Regner K. R., Dall A. T., Park F. (2018). Urinary exosomal expression of activator of G protein signaling 3 in polycystic kidney disease. *BMC Research Notes*.

[81] Salih M., Demmers J. A., Bezstarosti K. (2016). Proteomics of urinary vesicles links plakins and complement to polycystic kidney disease. *Journal of the American Society of Nephrology*.

[82] Bruschi M., Granata S., Santucci L. (2019). Proteomic analysis of urinary microvesicles and exosomes in medullary sponge kidney disease and autosomal dominant polycystic kidney disease. *Clinical Journal of the American Society of Nephrology*.

[83] Hogan M. C., Bakeberg J. L., Gainullin V. G. (2015). Identification of biomarkers for PKD1 using urinary exosomes. *Journal of the American Society of Nephrology*.

[84] Capitanio U., Bensalah K., Bex A. (2019). Epidemiology of renal cell carcinoma. *European Urology*.

[85] Zhao Y., Wang Y., Zhao E. (2020). PTRF/CAVIN1, regulated by SHC1 through the EGFR pathway, is found in urine exosomes as a potential biomarker of ccRCC. *Carcinogenesis*.

[86] Cravedi P., Kopp J. B., Remuzzi G. (2013). Recent progress in the pathophysiology and treatment of FSGS recurrence. *American Journal of Transplantation*.

[87] Huang Z., Zhang Y., Zhou J., Zhang Y. (2017). Urinary exosomal miR-193a can be a potential biomarker for the diagnosis of primary focal segmental glomerulosclerosis in children. *BioMed Research International*.

[88] Bhayana S., Song F., Jacob J. (2017). Urinary miRNAs as biomarkers for noninvasive evaluation of radiation-induced renal tubular injury. *Radiation Research*.

[89] Xie J. X., Fan X., Drummond C. A. (2017). MicroRNA profiling in kidney disease: plasma versus plasma-derived exosomes. *Gene*.

[90] Cho Y. E., Kim S. H., Lee B. H., Baek M. C. (2017). Circulating plasma and exosomal microRNAs as indicators of drug-induced organ injury in rodent models. *Biomolecules & Therapeutics*.

[91] Saejong S., Townamchai N., Somparn P. (2020). MicroRNA-21 in plasma exosome, but not from whole plasma, as a biomarker for the severe interstitial fibrosis and tubular atrophy (IF/TA) in post-renal transplantation. *Asian Pacific Journal of Allergy and Immunology*.

[92] Xiao C. T., Lai W. J., Zhu W. A., Wang H. (2020). MicroRNA derived from circulating exosomes as noninvasive biomarkers for diagnosing renal cell carcinoma. *Oncotargets and Therapy*.

[93] Dias F., Teixeira A. L., Nogueira I. (2020). Extracellular vesicles enriched in hsa-miR-301a-3p and hsa-miR-1293 dynamics in clear cell renal cell carcinoma patients: potential biomarkers of metastatic disease. *Cancers (Basel)*.

[94] Wang G., Yuan J., Cai X. (2020). HucMSC-exosomes carrying miR-326 inhibit neddylation to relieve inflammatory bowel disease in mice. *Clinical and Translational Medicine*.

[95] Yan Y., Jiang W., Tan Y. (2017). hucMSC exosome-derived GPX1 is required for the recovery of hepatic oxidant injury. *Molecular Therapy*.

[96] Shi H., Xu X., Zhang B. (2017). 3,3′-Diindolylmethane stimulates exosomal Wnt11 autocrine signaling in human umbilical cord mesenchymal stem cells to enhance wound healing. *Theranostics*.

[97] Jiang Y., Jahagirdar B. N., Reinhardt R. L. (2002). Pluripotency of mesenchymal stem cells derived from adult marrow. *Nature*.

[98] Zuk P. A., Zhu M., Ashjian P. (2002). Human adipose tissue is a source of multipotent stem cells. *Molecular Biology of the Cell*.

[99] Araújo A. B., Furlan J. M., Salton G. D. (2018). Isolation of human mesenchymal stem cells from amnion, chorion, placental decidua and umbilical cord: comparison of four enzymatic protocols. *Biotechnology Letters*.

[100] Hickson L. J., Eirin A., Lerman L. O. (2016). Challenges and opportunities for stem cell therapy in patients with chronic kidney disease. *Kidney International*.

[101] Peired A. J., Sisti A., Romagnani P. (2016). Mesenchymal stem cell-based therapy for kidney disease: a review of clinical evidence. *Stem Cells International*.

[102] Meng F., Xu R., Wang S. (2020). Human umbilical cord-derived mesenchymal stem cell therapy in patients with COVID-19: a phase 1 clinical trial. *Signal Transduction and Targeted Therapy*.

[103] Gu D., Zou X., Ju G., Zhang G., Bao E., Zhu Y. (2016). Mesenchymal stromal cells derived extracellular vesicles ameliorate acute renal ischemia reperfusion injury by inhibition of mitochondrial fission through miR-30. *Stem Cells International*.

[104] Zou X., Zhang G., Cheng Z. (2014). Microvesicles derived from human Wharton’s jelly mesenchymal stromal cells ameliorate renal ischemia-reperfusion injury in rats by suppressing CX3CL1. *Stem Cell Research & Therapy*.

[105] Zhang G., Zou X., Huang Y. (2016). Mesenchymal stromal cell-derived extracellular vesicles protect against acute kidney injury through anti-oxidation by enhancing Nrf2/ARE activation in rats. *Kidney & Blood Pressure Research*.

[106] Ju G. Q., Cheng J., Zhong L. (2015). Microvesicles derived from human umbilical cord mesenchymal stem cells facilitate tubular epithelial cell dedifferentiation and growth via hepatocyte growth factor induction. *PLoS One*.

[107] Zhou Y., Xu H., Xu W. (2013). Exosomes released by human umbilical cord mesenchymal stem cells protect against cisplatin-induced renal oxidative stress and apoptosis in vivo and in vitro. *Stem Cell Research & Therapy*.

[108] Zhang R., Zhu Y., Li Y. (2020). Human umbilical cord mesenchymal stem cell exosomes alleviate sepsis-associated acute kidney injury via regulating microRNA-146b expression. *Biotechnology Letters*.

[109] Liu B., Ding F., Hu D. (2018). Human umbilical cord mesenchymal stem cell conditioned medium attenuates renal fibrosis by reducing inflammation and epithelial-to-mesenchymal transition via the TLR4/NF-*κ*B signaling pathway in vivo and in vitro. *Stem Cell Research & Therapy*.

[110] Ji C., Zhang J., Zhu Y. (2020). Exosomes derived from hucMSC attenuate renal fibrosis through CK1*δ*/*β*-TRCP- mediated YAP degradation. *Cell Death & Disease*.

[111] Cao J. Y., Wang B., Tang T. T. (2021). Exosomal miR-125b-5p deriving from mesenchymal stem cells promotes tubular repair by suppression of p53 in ischemic acute kidney injury. *Theranostics*.

[112] Zhu F., Chong Lee Shin O. L. S., Pei G. (2017). Adipose-derived mesenchymal stem cells employed exosomes to attenuate AKI-CKD transition through tubular epithelial cell dependent Sox9 activation. *Oncotarget*.

[113] Gao F., Zuo B., Wang Y., Li S., Yang J., Sun D. (2020). Protective function of exosomes from adipose tissue-derived mesenchymal stem cells in acute kidney injury through SIRT1 pathway. *Life Sciences*.

[114] Li X., Liao J., Su X. (2020). Human urine-derived stem cells protect against renal ischemia/reperfusion injury in a rat model via exosomalmiR-146a-5pwhich targetsIRAK1. *Theranostics*.

[115] Zhang Y., Wang J., Yang B. (2020). Transfer of microRNA-216a-5p from exosomes secreted by human urine-derived stem cells reduces renal ischemia/reperfusion injury. *Frontiers in Cell and Development Biology*.

[116] Cao H., Cheng Y., Gao H. (2020). In VivoTracking of mesenchymal stem cell-derived extracellular vesicles improving mitochondrial function in renal ischemia-reperfusion injury. *ACS Nano*.

[117] Shen B., Liu J., Zhang F. (2016). CCR2 positive exosome released by mesenchymal stem cells suppresses macrophage functions and alleviates ischemia/reperfusion-induced renal injury. *Stem Cells International*.

[118] Jin J., Shi Y., Gong J. (2019). Exosome secreted from adipose-derived stem cells attenuates diabetic nephropathy by promoting autophagy flux and inhibiting apoptosis in podocyte. *Stem Cell Research & Therapy*.

[119] Ranghino A., Bruno S., Bussolati B. (2017). The effects of glomerular and tubular renal progenitors and derived extracellular vesicles on recovery from acute kidney injury. *Stem Cell Research & Therapy*.

[120] Pang P., Abbott M., Chang S. L. (2017). Human vascular progenitor cells derived from renal arteries are endothelial- like and assist in the repair of injured renal capillary networks. *Kidney International*.

[121] Viñas J. L., Burger D., Zimpelmann J. (2016). Transfer of microRNA-486-5p from human endothelial colony forming cell-derived exosomes reduces ischemic kidney injury. *Kidney International*.

[122] Zhou Y., Li P., Goodwin A. J. (2018). Exosomes from endothelial progenitor cells improve the outcome of a murine model of sepsis. *Molecular Therapy*.

[123] Zhang G., Zou X., Miao S. (2014). The anti-oxidative role of micro-vesicles derived from human Wharton-jelly mesenchymal stromal cells through NOX2/gp91(phox) suppression in alleviating renal ischemia-reperfusion injury in rats. *PLoS One*.

[124] Zhao M., Liu S., Wang C. (2021). Mesenchymal stem cell-derived extracellular vesicles attenuate mitochondrial damage and inflammation by stabilizing mitochondrial DNA. *ACS Nano*.

[125] Zhu G., Pei L., Lin F. (2019). Exosomes from human-bone-marrow-derived mesenchymal stem cells protect against renal ischemia/reperfusion injury via transferring miR-199a-3p. *Journal of Cellular Physiology*.

[126] Alzahrani F. A. (2019). Melatonin improves therapeutic potential of mesenchymal stem cells-derived exosomes against renal ischemia-reperfusion injury in rats. *American Journal of Translational Research*.

[127] Yuan X., Wang X., Chen C., Zhou J., Han M. (2017). Bone mesenchymal stem cells ameliorate ischemia/reperfusion-induced damage in renal epithelial cells via microRNA-223. *Stem Cell Research & Therapy*.

[128] Lin K. C., Yip H. K., Shao P. L. (2016). Combination of adipose-derived mesenchymal stem cells (ADMSC) and ADMSC- derived exosomes for protecting kidney from acute ischemia-reperfusion injury. *International Journal of Cardiology*.

[129] Ren Y., Chen Y., Zheng X. (2020). Human amniotic epithelial cells ameliorate kidney damage in ischemia-reperfusion mouse model of acute kidney injury. *Stem Cell Research & Therapy*.

[130] Wang B., Jia H., Zhang B. (2017). Pre-incubation with hucMSC-exosomes prevents cisplatin-induced nephrotoxicity by activating autophagy. *Stem Cell Research & Therapy*.

[131] Jia H., Liu W., Zhang B. (2018). HucMSC exosomes-delivered 14-3-3*ζ* enhanced autophagy via modulation of ATG16L in preventing cisplatin-induced acute kidney injury. *American Journal of Translational Research*.

[132] Wang J., Jia H., Zhang B. (2018). HucMSC exosome-transported 14-3-3*ζ* prevents the injury of cisplatin to HK-2 cells by inducing autophagy *in vitro*. *Cytotherapy*.

[133] Cao J., Wang B., Tang T. (2020). Three-dimensional culture of MSCs produces exosomes with improved yield and enhanced therapeutic efficacy for cisplatin-induced acute kidney injury. *Stem Cell Research & Therapy*.

[134] Reis L. A., Borges F. T., Simões M. J., Borges A. A., Sinigaglia-Coimbra R., Schor N. (2012). Bone marrow-derived mesenchymal stem cells repaired but did not prevent gentamicin-induced acute kidney injury through paracrine effects in rats. *PLoS One*.

[135] Tomasoni S., Longaretti L., Rota C. (2013). Transfer of growth factor receptor mRNA via exosomes unravels the regenerative effect of mesenchymal stem cells. *Stem Cells and Development*.

[136] Song Y., Dou H., Li X. (2017). Exosomal miR-146a contributes to the enhanced therapeutic efficacy of interleukin-1*β*-primed mesenchymal stem cells against sepsis. *Stem Cells*.

[137] Chang C. L., Sung P. H., Chen K. H. (2018). Adipose-derived mesenchymal stem cell-derived exosomes alleviate overwhelming systemic inflammatory reaction and organ damage and improve outcome in rat sepsis syndrome. *American Journal of Translational Research*.

[138] Liu B., Ding F. X., Liu Y. (2018). Human umbilical cord-derived mesenchymal stem cells conditioned medium attenuate interstitial fibrosis and stimulate the repair of tubular epithelial cells in an irreversible model of unilateral ureteral obstruction. *Nephrology (Carlton)*.

[139] Sheng L., Zhuang S. (2020). New insights into the role and mechanism of partial epithelial-mesenchymal transition in kidney fibrosis. *Frontiers in Physiology*.

[140] Fintha A., Gasparics Á., Rosivall L., Sebe A. (2019). Therapeutic targeting of fibrotic epithelial-mesenchymal transition-an outstanding challenge. *Frontiers in Pharmacology*.

[141] Li D., Zhang D., Tang B. (2019). Exosomes from human umbilical cord mesenchymal stem cells reduce damage from oxidative stress and the epithelial-mesenchymal transition in renal epithelial cells exposed to oxalate and calcium oxalate monohydrate. *Stem Cells International*.

[142] Chen L., Wang Y., Li S. (2020). Exosomes derived from GDNF-modified human adipose mesenchymal stem cells ameliorate peritubular capillary loss in tubulointerstitial fibrosis by activating the SIRT1/eNOS signaling pathway. *Theranostics*.

[143] Wang B., Jha J. C., Hagiwara S. (2014). Transforming growth factor-*β*1-mediated renal fibrosis is dependent on the regulation of transforming growth factor receptor 1 expression by let-7b. *Kidney International*.

[144] Wang B., Yao K., Huuskes B. M. (2016). Mesenchymal stem cells deliver exogenous microRNA-let7c via exosomes to attenuate renal fibrosis. *Molecular Therapy*.

[145] Ebrahim N., Ahmed I. A., Hussien N. I. (2018). Mesenchymal stem cell-derived exosomes ameliorated diabetic nephropathy by autophagy induction through the mTOR signaling pathway. *Cell*.

[146] Sun Y., Shi H., Yin S. (2018). Human mesenchymal stem cell derived exosomes alleviate type 2 diabetes mellitus by reversing peripheral insulin resistance and relieving *β*-cell destruction. *ACS Nano*.

[147] Li H., Rong P., Ma X. (2020). Mouse umbilical cord mesenchymal stem cell paracrine alleviates renal fibrosis in diabetic nephropathy by reducing myofibroblast transdifferentiation and cell proliferation and upregulating MMPs in mesangial cells. *Journal Diabetes Research*.

[148] Nagaishi K., Mizue Y., Chikenji T. (2016). Mesenchymal stem cell therapy ameliorates diabetic nephropathy via the paracrine effect of renal trophic factors including exosomes. *Scientific Reports*.

[149] Yu W., Zeng H., Chen J. (2020). miR-20a-5p is enriched in hypoxia-derived tubular exosomes and protects against acute tubular injury. *Clinical Science*.

[150] Chen H. H., Lai P. F., Lan Y. F. (2014). Exosomal ATF3 RNA attenuates pro-inflammatory gene MCP-1 transcription in renal ischemia-reperfusion. *Journal of Cellular Physiology*.

[151] Zhang L., Liu H., Xu K. (2019). Hypoxia preconditioned renal tubular epithelial cell-derived extracellular vesicles alleviate renal ischaemia-reperfusion injury mediated by the HIF-1*α*/Rab22 pathway and potentially affected by microRNAs. *International Journal of Biological Sciences*.

[152] Zhang W., Zhou X., Yao Q., Liu Y., Zhang H., Dong Z. (2017). HIF-1-mediated production of exosomes during hypoxia is protective in renal tubular cells. *American Journal of Physiology. Renal Physiology*.

[153] Viñas J. L., Spence M., Gutsol A. (2018). Receptor-ligand interaction mediates targeting of endothelial colony forming cell-derived exosomes to the kidney after ischemic injury. *Scientific Reports*.

[154] Tang T. T., Wang B., Wu M. (2020). Extracellular vesicle-encapsulated IL-10 as novel nanotherapeutics against ischemic AKI. *Science Advances*.

[155] Tapparo M., Bruno S., Collino F. (2019). Renal regenerative potential of extracellular vesicles derived from miRNA-engineered mesenchymal stromal cells. *International Journal of Molecular Sciences*.

[156] Grange C., Papadimitriou E., Dimuccio V. (2020). Urinary extracellular vesicles carrying klotho improve the recovery of renal function in an acute tubular injury model. *Molecular Therapy*.

[157] Pan T., Jia P., Chen N. (2019). Delayed remote ischemic preconditioning ConfersRenoprotection against septic acute kidney injury via exosomal miR-21. *Theranostics*.

[158] Wu P., Zhang B., Ocansey D. K. W., Xu W., Qian H. (2021). Extracellular vesicles: a bright star of nanomedicine. *Biomaterials*.

[159] Sato Y. T., Umezaki K., Sawada S. (2016). Engineering hybrid exosomes by membrane fusion with liposomes. *Scientific Reports*.

[160] Sun D., Zhuang X., Xiang X. (2010). A novel nanoparticle drug delivery system: the anti-inflammatory activity of curcumin is enhanced when encapsulated in exosomes. *Molecular Therapy*.

[161] Tang T. T., Lv L. L., Wang B. (2019). Employing macrophage-derived microvesicle for kidney-targeted delivery of dexamethasone: an efficient therapeutic strategy against renal inflammation and fibrosis. *Theranostics*.

[162] Yoon Y. M., Lee J. H., Song K. H., Noh H., Lee S. H. (2020). Melatonin-stimulated exosomes enhance the regenerative potential of chronic kidney disease-derived mesenchymal stem/stromal cells via cellular prion proteins. *Journal of Pineal Research*.

[163] Zhang C., Shang Y., Chen X. (2020). Supramolecular nanofibers containing arginine-glycine-aspartate (RGD) peptides boost therapeutic efficacy of extracellular vesicles in kidney repair. *ACS Nano*.

[164] Liu Y., Cui J., Wang H. (2020). Enhanced therapeutic effects of MSC-derived extracellular vesicles with an injectable collagen matrix for experimental acute kidney injury treatment. *Stem Cell Research & Therapy*.

[165] Park K. S., Svennerholm K., Shelke G. V. (2019). Mesenchymal stromal cell-derived nanovesicles ameliorate bacterial outer membrane vesicle-induced sepsis via IL-10. *Stem Cell Research & Therapy*.

[166] Liu X., Miao J., Wang C. (2020). Tubule-derived exosomes play a central role in fibroblast activation and kidney fibrosis. *Kidney International*.

[167] Lv L. L., Feng Y., Wen Y. (2018). Exosomal CCL2 from tubular epithelial cells is critical for albumin-induced tubulointerstitial inflammation. *Journal of the American Society of Nephrology*.

[168] Li Z. L., Lv L. L., Tang T. T. (2019). HIF-1*α* inducing exosomal microRNA-23a expression mediates the cross-talk between tubular epithelial cells and macrophages in tubulointerstitial inflammation. *Kidney International*.

[169] Munkonda M. N., Akbari S., Landry C. (2018). Podocyte-derived microparticles promote proximal tubule fibrotic signaling via p38 MAPK and CD36. *Journal of Extracellular Vesicles*.

[170] Guan H., Peng R., Mao L., Fang F., Xu B., Chen M. (2020). Injured tubular epithelial cells activate fibroblasts to promote kidney fibrosis through miR-150-containing exosomes. *Experimental Cell Research*.

[171] Wang Y. Y., Tang L. Q., Wei W. (2018). Berberine attenuates podocytes injury caused by exosomes derived from high glucose-induced mesangial cells through TGF*β*1-PI3K/AKT pathway. *European Journal of Pharmacology*.

[172] Wu X. M., Gao Y. B., Cui F. Q., Zhang N. (2016). Exosomes from high glucose-treated glomerular endothelial cells activate mesangial cells to promote renal fibrosis. *Biology Open*.

[173] Wu X., Gao Y., Xu L. (2017). Exosomes from high glucose-treated glomerular endothelial cells trigger the epithelial-mesenchymal transition and dysfunction of podocytes. *Scientific Reports*.

[174] Gimona M., Brizzi M. F., Choo A. B. H. (2021). Critical considerations for the development of potency tests for therapeutic applications of mesenchymal stromal cell-derived small extracellular vesicles. *Cytotherapy*.

[175] Wu Z., He D., Li H. (2021). Bioglass enhances the production of exosomes and improves their capability of promoting vascularization. *Bioactive Materials*.

[176] Grange C., Skovronova R., Marabese F., Bussolati B. (2019). Stem cell-derived extracellular vesicles and kidney regeneration. *Cell*.

[177] Lv L. L., Wu W. J., Feng Y., Li Z. L., Tang T. T., Liu B. C. (2017). Therapeutic application of extracellular vesicles in kidney disease: promises and challenges. *Journal of Cellular and Molecular Medicine*.

[178] Zhou W., Zhou Y., Chen X. (2021). Pancreatic cancer-targeting exosomes for enhancing immunotherapy and reprogramming tumor microenvironment. *Biomaterials*.

[179] Teng F., Fussenegger M. (2021). Shedding light on extracellular vesicle biogenesis and bioengineering. *Advanced Science*.

